# Peripheral perfusion index of pulse oximetry in adult patients: a narrative review

**DOI:** 10.1186/s40001-024-02048-3

**Published:** 2024-09-11

**Authors:** Xiaotong Sun, Huaiwu He, Mengru Xu, Yun Long

**Affiliations:** State Key Laboratory of Complex Severe and Rare Diseases, Department of Critical Care Medicine, Peking Union Medical College Hospital, Peking Union Medical College, Chinese Academy of Medical Science, Beijing, China

**Keywords:** Peripheral perfusion index, Pulse oximetry, Shock, Critical care/emergency medicine

## Abstract

The peripheral perfusion index (PI) is derived from pulse oximetry and is defined as the ratio of the pulse wave of the pulsatile portion (arteries) to the non-pulsatile portion (venous and other tissues). A growing number of clinical studies have supported the use of PI in various clinical scenarios, such as guiding hemodynamic management and serving as an indicator of outcome and organ function. In this review, we will introduce and discuss this traditional but neglected indicator of the peripheral microcirculatory perfusion. Further clinical trials are required to clarify the normal and critical values of PI for different monitoring devices in various clinical conditions, to establish different standards of PI-guided strategies, and to determine the effect of PI-guided therapy on outcome.

## Introduction

Pulse oximetry has been widely used in clinical practice. The pulse waveform recorded by photoplethysmography could provide information on tissue perfusion using changes in light transmission with changes in blood volume within the tissue [1, 2]. The peripheral perfusion index (PI) was derived from the peripheral pulse waveform, defined as the ratio of the pulse wave of the pulsatile portion to the non-pulsatile portion. PI reflects the change in blood volume with each heartbeat in the fingers. It is easy to measure and could be displayed continuously on the monitor. PI works as a ratio without a unit, and it does not measure direct tissue perfusion. In contrast to the SpO_2_, the PI has traditionally been neglected. However, the interest of using PI to assess peripheral microcirculatory perfusion has brought it to the forefront of critical care medicine. Numerous clinical studies had shown that normalized macro-circulatory parameters could not guarantee the restoration of microcirculatory perfusion [3, 4], and attention has been paid on these microcirculatory perfusion targets during shock resuscitation. New technologies and parameters for microcirculation assessment have undergone great development, such as sublingual microcirculation by side-stream dark-field (SDF) imaging [5], tissue oxygen saturation [6] and transcutaneous partial pressure of oxygen [6], providing much insight into accurate assessment of microcirculation. PI is also considered a promising indicator of peripheral microcirculation. Many studies have found that PI can provide useful information for shock resuscitation [7], fluid management [8], vasopressor therapy [9], outcome prediction [10, 11], risk stratification [12], and pain assessment [13]. In this narrative review, we reviewed the literatures on the use of PI in different clinical conditions, explored the reference range, revealed potential benefits, and summarized the challenge and future research direction of PI in critically ill adult patients.

## Measurement and reference range of PI

### (1) Measurement principle

The pulse oximetry probe generates ultra-red light beams whose transmitted intensities are converted into an electrical current by a photodetector after passing through tissue. The signal received by the photodetector is then separated into pulsatile and non-pulsatile signals. The pulsatile signal represents variations in light absorption due to pulsatile vessels under variations in arterial pressure. It is an indirect measurement of arterial volume variation during the cardiac cycle. Non-pulsatile signal is the continuous light absorption from non-pulsatile capillaries, venous vessels, skin, soft tissue and bone. PI is the ratio of pulsatile to non-pulsatile light absorption of the photoplethysmography signal.

### (2) Measurement method

It is important for intensivists to obtain an accurate PI value before using PI to guide therapy at the bedside. These following factors that affect accurate signal acquisition should be excluded: device connection, nail polish, ambient light, motion artifacts caused by spontaneous movement [14]. For the measurement site, PI can be obtained from fingers, toes, forehead, earlobe, etc. The middle finger is the most common site for PI monitoring in clinical trials and should be considered the standard site for PI monitoring. One study found a similar trend in obtaining PI through the fingers, forehead and earlobe of 29 adult patients undergoing surgery [15]. Moreover, the PI value varies on different fingers [16]. In healthy adults, Swain et al. [17] found the highest PI was obtained via the middle finger, while Sapra et al. [18] recorded the maximal PI via the right-hand ring finger. Further investigations are required to validate the relevance of obtaining PI at different measurement sites. Furthermore, individual variations in tissue edema and differences in finger size should be taken into account when interpreting the PI value.

### (3) Measurement determinants and potential impact factors

**Fig. 1:**
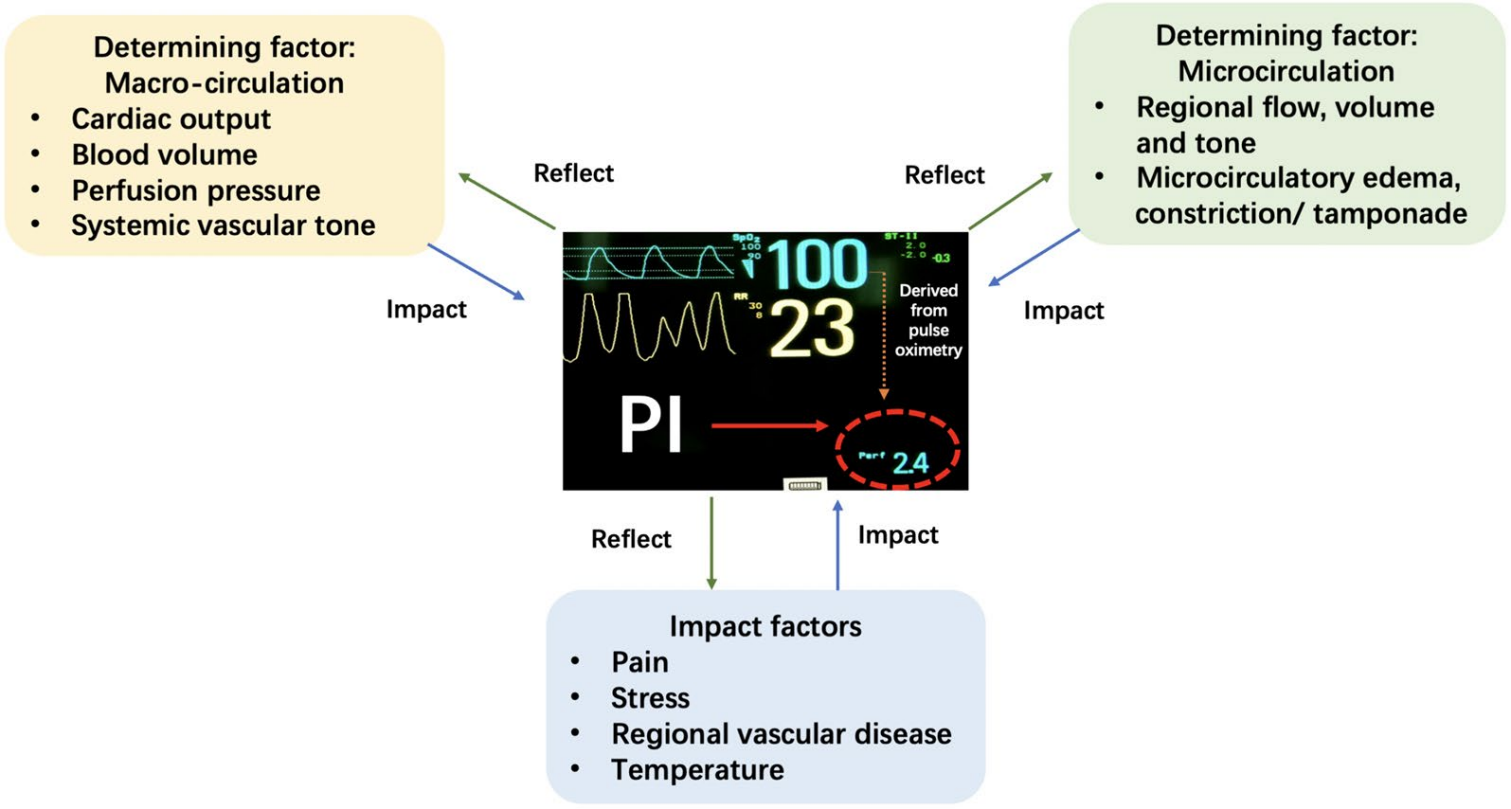
Determinants and impact factors of PI. Determinants of PI include cardiac output [25], blood volume [26], perfusion pressure, vascular tone [20], microcirculation failure [27]. Impact factors of PI include pain [22], stress [13], peripheral vascular diseases [19] and body temperature [28]

Two main determinants of PI are macro-circulation and regional microcirculation. Macro-circulation dysfunction, such as hypovolemia, low cardiac output (CO) and abnormal vascular tone, could directly lead to an impaired PI. Moreover, microcirculation failure after the correction of macro-circulation could result in a low PI. In addition, many other factors such as peripheral vascular diseases, body temperature, pain and stress could impact PI [19–21]. Therefore, both main determinants and other impact factors mentioned above need to be taken into account when interpreting PI. Figure 1 summarizes the impact factors of PI. In addition, studies have shown that gender, age, weight and body position can influence PI values [22–24].

### (4) PI reference range in different populations

PI has high interindividual variability, and its distribution is skewed in healthy volunteers and critically ill patients [29]. Lima et al. [29] showed that PI was 1.4 (0.7–3.0), but another study found PI was 3.9 (2.9–6.1) in the healthy adults [30]. Different measuring devices and populations might explain the different reference range. Compared to the healthy adults, critically ill patients had a lower PI value [31–33]. Moreover, the reference range varied among critically ill patients with different diseases. The median PI was 1.3 in patients with shock [31], and the PI was 1.2 in the patients with pre-hospital return of spontaneous circulation (ROSC) after out-of-hospital cardiac arrest (OHCA) [32]. In addition, PI was found to be 0.8 and 0.7 in in survivors and non-survivors under therapeutic hypothermia to 33 °C after OHCA, respectively [33]. The PI reference range reported in different populations are summarized in Table 1.

**Table 1:** PI values in different populations

Study population		Age, yr median (IQR)	Measurement site	PI value median (IQR) /mean (SD)
Healthy adults (*n* = 108) [29]		36 (30–45)	Fingers	1.4 (0.7–3.0)
Healthy adults (*n* = 180) [30]		32 (21–39)	Fingers	3.9 (2.9–6.1)
Patients with circulatory shock (*n* = 70) [31]		63 (53–73)	Fingers	1.3 (0.5–2.1)
Patients with ROSC after OHCA (*n* = 164) [32]		70 (58.5–78)	Fingers	1.2 (0.6–2.38)
Patients with therapeutic hypothermia (33 °C) after OHCA [33]				
Survivors (*n* = 19)		59 (57–76)	Fingers	0.8 (0.13)
Nonsurvivors (*n* = 6)		71 (66–79)		0.7 (0.23)

*PI* peripheral perfusion index, *IQR* interquartile range, *SD* standard deviation, *ROSC* return of spontaneous circulation, *OHCA* out-of-hospital cardiac arrest

If a single PI value is below the critical reference value, it could be taken as an early warning indicator of low tissue perfusion. It should be noted, however, that it is difficult to evaluate PI as an absolute value. PI is a ratio without units and must be evaluated on a relative basis. Hence, pursuing a higher PI value could not guarantee a good perfusion in some special conditions. For example, patients with a PI of 1 usually have better perfusion than patients with a PI of 0.1. However, a PI of 10 is not necessarily better than a PI of 6 in patients with aortic regurgitation. The high PI could be caused by a high pulse pressure in this condition, where tissue perfusion is not necessarily good. Mongkolpun et al. [31] also found that capillary refill time (CRT) and skin laser Doppler performed better than PI in predicting outcome in patients with circulatory shock. The authors found some shock patients had a PI > 1.4. Hence, combining PI with other perfusion parameters is helpful in making a comprehensive decision about tissue perfusion.

## Applications for hemodynamic management

Since macro-circulation and microcirculation could impact PI value, the PI is used to reflect macro-circulatory and microcirculatory related contents.

### (1) Assessment of fluid response and hypovolemia

As PI has been shown to reflect CO and the regional blood volume [34], an increase in PI after a rapid fluid infusion or passive leg raising (PLR) test might indicate the presence of fluid response. Studies using PI to predict fluid response are summarized in Table 2. In patients with septic shock, a 33% increase in PI after infusing 250 mL to 750 mL of crystalloid over 30 min [34] or a 5% increase in PI after infusing 200 mL of crystalloid over 1 min could predict fluid response [35]. Besides, a PLR-induced increase in PI > 9% reliably detected a positive PLR test in patients with shock [25]. Concerning ventilated patients, methods using heart–lung interactions are feasible to identify fluid responders. For example, a lung recruitment maneuver-induced decrease in PI ≥ 26% was predictive of a decrease in the stroke volume ≥ 30% [36], and an increase in PI > 2.5% during the end-expiratory occlusion test could detect a positive PLR test [37]. A large variation in the PI cutoff value (from 2.5% to 33%) might be due to different methods of assessing fluid response. In addition, the correlation between PI and cardiac index (CI) was not good and varied between studies (r value of PI and CI ranged from 0.39 to 0.83). Further studies with larger samples are required to determine the cutoff value for using the change in PI to predict fluid response under different conditions.

**Table 2:** PI in fluid response prediction

Study population	Methods of fluid response evaluation	Fluid responders	Predictive cutoff value of PI	AUROC (95% CI)
Patients with septic shock (*n* = 55) [34]	250 mL to 750 mL fluid challenge	Increase in cardiac index > 10%	∆PI > 33%	0.78 (0.65–0.91)
Patients with septic shock (*n* = 58) [35]	200 mL fluid challenge	Echocardiography-derived increase in VTI > 10% after 500 mL fluid infusion	∆PI > 5%	0.82 (0.70–0.91)
Patients with acute circulatory failure (*n* = 72) [25]	PLR test	A PLR-induced increase in cardiac index ≥ 10%	∆PI > 9%	0.89 (0.80–0.95)
Patients with mechanical ventilation (*n* = 31) [37]	End-expiratory occlusion test	A PLR-induced increase in cardiac index ≥ 10%	∆PI > 2.5%	1.00 (1.00–1.00) in patients with baseline PI ≤ 1 0.93 (0.81–1.00) in patients with baseline PI > 1
Ventilated patients undergoing neurosurgery (*n* = 47) [36]	Lung recruitment maneuver	Decrease in stroke volume ≥ 30%	∆PI ≥ 26%	0.84 (0.71–0.93)

*PI* peripheral perfusion index, *AUROC* area under receiver operating characteristic curve, *CI* confidence interval, *VTI* velocity time integral, *PLR* passive leg raising, ∆PI = [PI value at the end of fluid response evaluation–PI value before evaluation]/PI value before evaluation × 100

Moreover, a low PI was taken as an indicator of hypovolemia during the negative fluid balance treatment. In patients with acute kidney injury, a low baseline PI could predict hypotension during fluid removal by renal replacement therapy [38, 39]. As a low baseline PI reflects high sympathetic activity and peripheral vasoconstriction [40], it is difficult for the vessels to constrict further during dialysis-induced hypovolemia. It is suggested that intensivists should reduce the rate and amount of fluid removal during renal replacement therapy in patients with low baseline PI.

### (2) Combined with macro-circulation for fluid management during resuscitation

PI is of potential interest for initiating/terminating fluid resuscitation and negative fluid balance. Poor PI could trigger fluid resuscitation and fluid response should be suspected in the salvage and optimization phases of circulatory shock. When PI indicates satisfactory tissue perfusion and no fluid response, intensivists should stop resuscitation and consider removal of excess fluid. The study by van Genderen et al. [7] showed that patients with septic shock received less fluid when peripheral perfusion parameters were used to guide resuscitation. Moreover, the peripheral perfusion-guided group had a shorter hospital stay and lower organ failure scores than the lactate-guided group. The combination of PI with macro-circulation indicators such as central venous oxygen saturation (ScvO_2_) helps to provide individualized hemodynamic management. Based on PI and ScvO_2_, tissue perfusion can be divided into the following four types [8]: type 1 (PI < 0.6 on ScvO2 < 70%), type 2 (PI < 0.6 on ScvO2 > 70%), type 3 (PI > 0.6 on ScvO2 < 70%), type 4 (PI > 0.6 on ScvO2 > 70%). The first type suggests that tissue perfusion can be improved by improving macro-circulation. In the second type, therapy should focus on the damage caused to the microcirculation by the primary disease, such as inadequate infection control. In the third type, dynamic assessment in combination with other perfusion indicators should be applied since the microcirculation has recovered. The fourth type suggests that reverse volume resuscitation should be started and further recovery of organ function should be considered. Future studies could explore the combination of PI and other hemodynamic indicators such as lactate for resuscitation, which may be helpful in interpreting the coherence of microcirculation and cellular oxygen metabolism.

### (3) Assessment of vascular tone

Vascular tone refers to the extent of constriction of blood vessels relative to their maximal dilated state. Vasoactive drugs, anesthesia and pain can cause changes in vascular tone. In general, PI is negatively correlated with vascular tone. In surgical patients, an increase in PI induced by local anesthetic injection may be an early indicator of successful regional nerve blocks [41]. Besides, patients with high PI values may be more likely to develop hypotension after anesthesia due to vasodilation. For example, parturients have low systemic vascular resistance. Before cesarean section, parturients with a baseline PI > 3.5 were expected to have lower peripheral vascular tone and were at higher risk of developing hypotension after spinal anesthesia [42]. Norepinephrine could lead to vasoconstriction, which could cause a change in PI. However, in some cases the relationship between vascular tone and PI is complex and non-linear. Rasmy et al. [9] found a decrease in PI with the use of norepinephrine for normal MAP in patients with septic shock. Our previous study [43] found that with increasing norepinephrine infusion there was significant change in MAP during norepinephrine titration. However, there was no significant and consistent change in continuous CO and PI at different MAP levels. It was suggested that PI may have potential applications for optimizing vasopressor therapy based on changes in peripheral tissue perfusion in septic shock patients.

## Prediction of outcome and *indicator* of organ function

Numerous studies have found PI had potential interest in prediction of outcome and organ function in critically ill patients.

### (1) Prediction of outcome

PI, as a surrogate for peripheral microcirculation, has also been found to be a valuable predictor of severity and prognosis in critically ill patients. Studies using PI to predict outcome in different types of patients are shown in Table 3.

**Table 3:** PI in outcome prediction in different kinds of patients

Study population	Age, yr median (IQR) /mean (SD)	Outcome	Predictive cutoff value of PI	AUROC (95% CI)
Patients with tissue hypoperfusion (*n* = 37) [29]	70 (13)	Poor peripheral perfusion	PI < 1.4	0.91 (0.84–0.98)
Patients with tissue hypoperfusion (*n* = 202) [8]	57 (18)	30-day mortality	PI < 0.6	0.84 (0.78–0.88)
Patients with sepsis (*n* = 46) [10]	62 (16)	ICU mortality	PI ≤ 0.2	0.84 (0.70–0.93)
Patients with sepsis (*n* = 36) [9]	50 (18)	28‐day mortality	PI ≤ 0.21	0.94 (0.8–0.99)
Patients with OHCA (*n* = 164) [32]	70 (59–78)	30-day mortality or poor neurologic outcome	MPI_30_ was an independent predictor with an RR of 0.85 (0.72–0.99)	
Patients with mechanical ventilation (*n* = 5,103) [11]	61 (48–72) in survivors 61 (52–72) in nonsurvivors	ICU mortality	PI < 1.37	0.76 (0.21–0.27)
Surgical patients (*n* = 168) [49]	55 (11) in PG 57 (11) in nPG	ICU stay > 48 h	PI < 1.35	0.77 (0.66–0.89)

*PI* peripheral perfusion index, *IQR* interquartile range, *SD* standard deviation, *AUROC* area under receiver operating characteristic curve, *CI* confidence interval, *OHCA* out-of-hospital cardiac arrest, *MPI*_*30*_ the mean value of the PI over 30 min after ROSC, *RR* Relative Risk, *PG* prolonged group in which patients stayed in ICU longer than 48 h, *nPG* non prolong group in which patients stayed in ICU shorter than 48 h

*Patients with shock* Our previous study found that a PI < 0.6 after resuscitation was predictive of 30-day mortality [8] and a PI ≤ 0.2 after resuscitation was predictive of ICU mortality [10]. The study by Rasmy et al. also found that a PI ≤ 0.2 could predict 28-day mortality [9]. In addition, Pan et al. [44] and de Miranda et al. [45] showed that a lower PI was associated with a higher risk of organ dysfunction and 28‐day mortality in patients with septic shock and sepsis-associated acute kidney injury. In patients with non-septic shock, Valle ´e et al. [46] found that the heat challenge-induced increase in PI was significantly greater in survivors than in non-survivors on the second day of hospitalization. This reflected that non-survivors had impaired vasoreactivity. In summary, a low PI has been proven to be an indicator of poor outcome in patients with shock.

*Patients with OHCA* Patients resuscitated from an OHCA have poor peripheral perfusion. Savastano et al. [32] reported that the mean value of PI in 30 min after ROSC could independently predict 30-day mortality and brain injury in patients with OHCA. The study by van Genderen et al. [33] also showed that PI was significantly lower in nonsurvivors after rewarming from therapeutic hypothermia in patients with OHCA.

*Patients with mechanical ventilation *PI is an early predictor of prognosis in ventilated patients. Su et al. [11] found that a PI < 1.37 during the first 24 h after ICU admission was a good predictor of in-ICU mortality. Er et al. [47] also found that PI at 24 h after ICU admission was independently correlated with 7-day mortality.

*Surgical patients* Research has shown that a PI < 1.4 on the second day after surgery is predictive of severe postoperative complications independent of systemic hemodynamics [48]. It also found that the CRT appeared to alter from the immediate postoperative period and showed better performance. In addition, a PI < 1.35 within the first 6 h of ICU admission could predict an ICU stay longer than 48 h [49], earlier and more accurately than lactate.

### (2) *Indicator* of organ function

PI, as an indicator of finger microcirculation, has some relationship with organ perfusion and function in critically ill patients. Studies found a low PI was associated with a high SOFA score [44, 50]. In patients with septic shock, the highest SOFA score (14.5 ± 2.9) was found in the low PI and ∆PPV (perfusion vessel change rate derived from sublingual microcirculation monitoring) group [44]. As for patients with sepsis, Guo et al. [51] showed that PI was negatively associated with coagulation markers (prothrombin time and activated partial thromboplastin time) and a marker of myocardial injury (cardiac troponin I), suggesting a potential association between PI and organ function. However, Miranda et al. [45] found no difference in PI between septic patients with and without acute kidney injury. The authors attributed the result to the different microcirculation structures and local homeostasis of the renal and skin. Few studies focus on the direct correlation between PI and microcirculation in each visceral organ. One of the reasons may be the difficulty in assessing visceral blood flow. Doppler sonography [52] and orthogonal polarization spectral imaging [53] may be useful in assessing visceral organ perfusion. Further studies are needed to explore the relationship between PI and the microcirculation of each organ in different critical diseases and stages.

## Other clinical applications of PI

There are other potential applications of PI in the clinical practice. The relevant content and literature are summarized as follows.

*(1) Prediction of successful ventilator weaning* Clinical study had shown that an increase in PI of more than 41% during the spontaneous breathing test could predict successful weaning [54]. This could be explained by increased CO during spontaneous breathing as intrathoracic pressure decreases and venous return increases.

*(2) Indicator in pain assessment* Painful stimuli could activate the sympathetic nervous system and increase vascular tone, leading to a decrease in PI. PI has, therefore, been proposed to assess pain in critically ill patients who are unable to express themselves. Hasanin et al. [13] found that a decrease in PI > 0.7 had a good ability to predict an increase of three points in the behavioral pain scale score in non-intubated patients after pain stimulation. In intubated patients, Abdelhakeem et al. [55] found a small but significant negative correlation between the change in PI and the change in the behavioral pain scale score. Therefore, PI could be a convenient indicator to systematically assess pain, which has been shown to be associated with reduced duration of mechanical ventilation [56, 57].

*(3) Assessment of the accuracy of SpO*_*2*_* and glucose measurement* Poor peripheral perfusion might affect the accuracy of measurements such as SpO_2_ and capillary blood glucose (CBG). PI can potentially be used to detect the measurement error of these parameters. SpO_2_ measured by pulse oximetry is more likely to be inaccurate in patients with poor perfusion [58]. Louie et al. [59] found that a PI < 2 was related to increased bias in SpO_2_ and arterial oxygen saturation on three types of pulse oximeters. For CBG, Desachy et al. [60] found that a low PI was independently associated with poor capillary glucose test strip performance. The accuracy of the point-of-care testing, including SpO_2_ and CBG, was impaired in a low PI condition. Therefore, arterial blood gas and whole blood glucose testing are more recommended in critically ill patients with low PI.

*(4) Identify false-positive ECG for ST-segment elevation myocardial infarction in patients with ROSC* A study showed that a lower PI value within 30 min after ROSC was significantly associated with a higher rate of false-positive ECG for ST-segment elevation myocardial infarction [61]. In patients with a normal PI after ROSC, the ST-segment elevation recorded by electrocardiogram (ECG) may reflect myocardial ischemia caused by the coronary artery obstruction. In patients with a low PI after ROSC, the ST-segment elevation recorded by ECG may reflect myocardial ischemia caused by the low coronary artery flow. The coronary angiography did not show significant coronary stenosis in this situation. Hence, it is encouraged to perform another ECG when PI increases to identify patients who may benefit from urgent coronary angiography.

*(5) Indicator of risk stratification in different clinical conditions* In emergency departments, a 1-point decrease in PI would increase the likelihood of hospitalization by 29% [12]. In patients with pulmonary embolism, PI might be helpful in predicting mortality and the need for mechanical ventilation, inotropic treatment and thrombolytic therapy [62]. In addition, a PI < 1 and PI < 1.17 are good indicators of the need for blood transfusion in patients with multi-trauma and upper gastrointestinal system bleeding, respectively [63, 64].

## Challenges and future directions

### (1) Challenges in clinical applications

PI is a promising non-invasive bedside indicator of peripheral perfusion, but it is sometimes neglected. The reasons are various. First, many factors such as pain [22], peripheral vascular disease [19] and body temperature [28] could affect the PI value, making data interpretation difficult. Second, the cutoff value of PI was changed in different conditions, and relative inter-individual variation was present. The distribution of PI is skewed in healthy adults, ranging from 0.3 to 10 [29], and the threshold varies in critically ill patients with different diseases, as shown in Table 1. These features could easily be mistaken for the PI measurement issue of accuracy. Third, different algorithms of PI in different monitoring devices could further cause the basis of PI value. For example, some devices try to identify and eliminate the motion artifacts using adaptive filters and secondary sensors, which could reduce the error in PI measurements [65]. Fourth, more attention is paid to pulse oximetry based on traditional clinical thinking. The relevance of using the SpO_2_ waveform to distinguish an artifact from the true signal has been emphasized, and low perfusion is taken as one limitation for pulse oximetry [59].

### (2) Future directions

With the aim to explore the clinical applications of PI, the following research topics are highlighted in the future.

#### (1) Definition of PI normal and critical values

Sacrifice of peripheral perfusion is a self-protective mechanism, so impairment of peripheral perfusion may be acceptable to some extent. In contrast, normalization of tissue perfusion may be an indicator of fluid de-resuscitation. A "mildly impaired peripheral perfusion" may be permissive and does not require immediate and aggressive resuscitation [66]. Moreover, there are different machines and calculated formula for PI monitoring. Hence, the normal and critical values of PI should be determined based on a large sample population for healthy volunteers and different critical illness conditions in different devices.

#### (2) Standards of PI-guided strategy

**Fig. 2:**
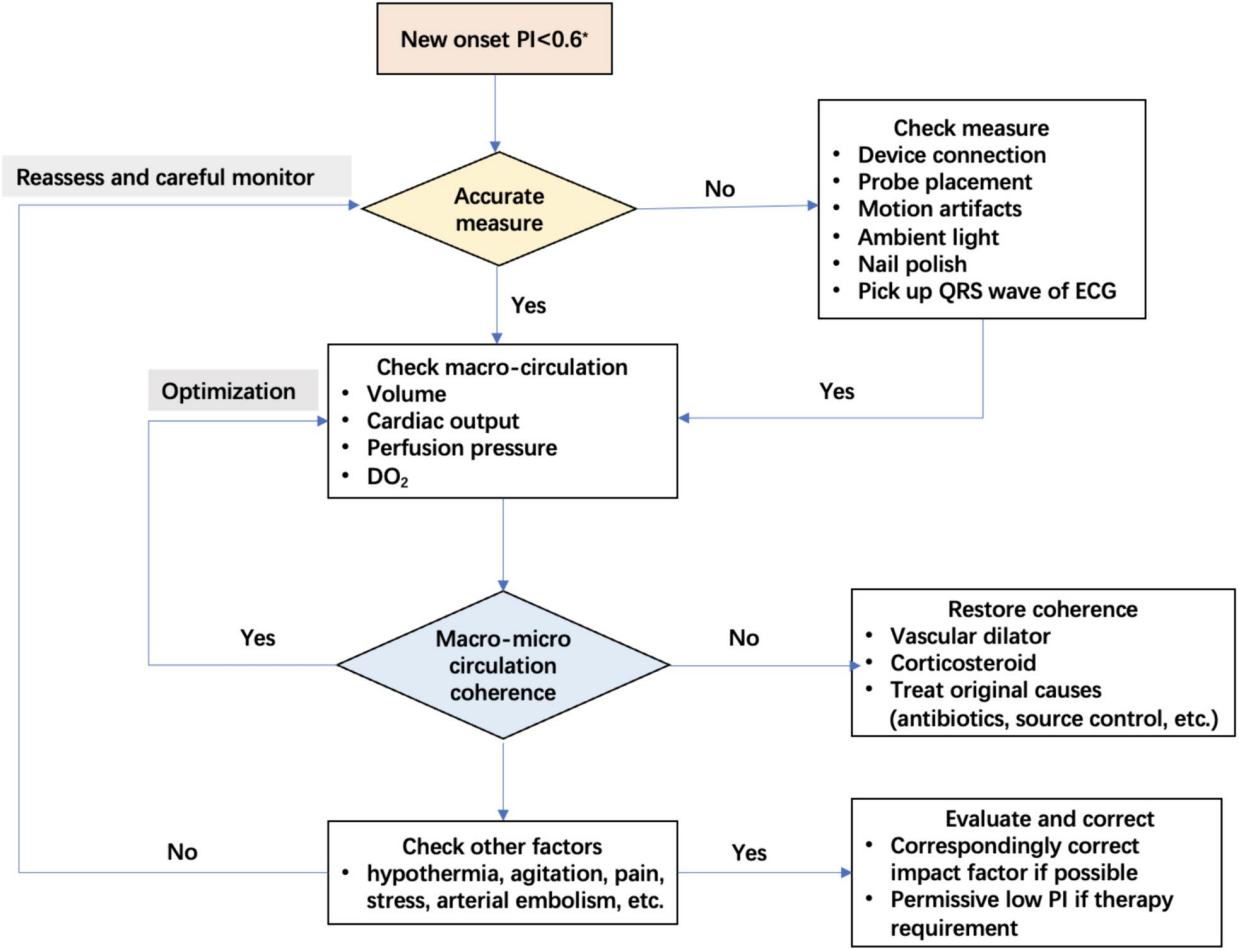
Proof of concept to interpret and manage a low PI in critically ill adults. *PI < 0.6 was referred to our previous research [8] *PI* peripheral perfusion index, *ECG* electrocardiogram, *CVP* central venous pressure, *CO* cardiac output, *ScvO*_*2*_ central venous oxygen saturation, *MAP* mean arterial pressure, *PE* pulmonary embolism, *CaO*_*2*_ arterial oxygen content, *SaO*_*2*_ arterial oxygen saturation

Clinical decision tree of PI deserves to be summarized and validated in different clinical conditions. Moreover, potential impact factors of PI such as temperature, level of consciousness, pain and other stress stimuli, endogenous catecholamines and vasopressors could be considered in a complex mode to interpret a low PI in the future. With the aim to improve the understanding of PI at the bedside, a protocol for the management of low PI was summarized based on the potential benefit of PI and the impact factors (Fig. 2). We chose 0.6 as the threshold based on the experience of our hospital and the result of our previous study which showed that PI < 0.6 was a risk factor for adverse outcome in critically ill patients. The generalizability of this threshold needs to be explored in further experiments. Further studies are required to validate this protocol.

#### (3) Effect of PI-guide management on outcome

In the ANDROMEDA-Shock study, a resuscitation strategy targeting normalization of CRT (< 3 s) did not reduce 28-day all-cause mortality compared with a strategy targeting serum lactate levels [67]. PI may have the advantage of real-time monitoring over the manual measurement of CRT. Hence, clinical trials should be conducted to confirm the influence of serial strategies of PI-guided therapy on patient outcome. PI-guided strategies could include fluid management (resuscitation and de-resuscitation) and vasopressor titration.

## Conclusion

As a noninvasive and objective indicator of peripheral tissue perfusion, PI has been shown to be useful in many aspects in critically ill patients. This review summarizes its applications in hemodynamic management (fluid resuscitation, de-resuscitation and vasopressor therapy) and prediction of outcome and organ function in critically ill patients. The factors influencing PI should be considered when interpreting a low PI. Further research should focus on the effect of PI-guided therapy on outcomes.

## Data Availability

Not applicable.

## Abbreviations

SpO_2_: Pulse oxygen saturation percentage
PI: Peripheral perfusion index
CO: Cardiac output
ROSC: Return of spontaneous circulation
OHCA: Out-of-hospital cardiac arrest
ICU: Intensive care unit
PLR: Passive leg raising
VTI: Velocity time integral
MAP: Mean arterial pressure
ScvO_2_: Central venous oxygen saturation
MPI_30_: The mean value of the PI over 30 min after ROSC
PG: Prolonged group in which patients stayed in ICU longer than 48 h
nPG: Non prolong group in which patients stayed in ICU shorter than 48 h
CBG: Capillary blood glucose
ECG: Electrocardiogram
STEMI: ST-segment elevation myocardial infarction
CVP: Central venous pressure
PE: Pulmonary embolism
CaO_2_: Arterial oxygen content
SaO_2_: Arterial oxygen saturation
